# Exploring genetic loci linked to COVID-19 severity and immune response through multi-trait GWAS analyses

**DOI:** 10.3389/fgene.2025.1502839

**Published:** 2025-02-17

**Authors:** Ziang Meng, Chumeng Zhang, Shuai Liu, Wen Li, Yue Wang, Qingyi Zhang, Bichen Peng, Weiyi Ye, Yue Jiang, Yingchao Song, Miao Guo, Xiao Chang, Lei Shao

**Affiliations:** ^1^ Department of Infectious Disease, Central Hospital Affiliated to Shandong First Medical University, Jinan, Shandong, China; ^2^ The Second School of Clinical Medicine of Shandong First Medical University, Tai’an, Shandong, China; ^3^ Agricultural Products Quality and Safety Center of Jinan, Jinan, Shandong, China; ^4^ College of Medical Information and Artificial Intelligence, Shandong First Medical University, Jinan, Shandong, China; ^5^ School of Life Sciences, Shandong First Medical University, Shandong, China

**Keywords:** COVID-19, lymphocyte, immune response, genome-wide cross-trait analysis, transcriptome-wide association studies

## Abstract

**Introduction:**

COVID-19 severity has been linked to immune factors, with excessive immune responses like cytokine storms contributing to mortality. However, the genetic basis of these immune responses is not well understood. This study aimed to explore the genetic connection between COVID-19 severity and blood cell traits, given their close relationship with immunity.

**Materials and methods:**

GWAS summary statistics for COVID-19 and blood cell counts were analyzed using Linkage Disequilibrium Score Regression (LDSC) to estimate genetic correlations and heritabilities. For traits with significant correlations, a Multi-Trait GWAS Analysis (MTAG) was performed to identify pleiotropic loci shared between COVID-19 and blood cell counts.

**Results:**

Our MTAG analysis identified four pleiotropic loci associated with COVID-19 severity, five loci linked to hospitalized cases, and one locus related to general patients. Among these, two novel loci were identified in the high-risk population, with rs55779981 located near *RAVER1* and rs73009538 near *CARM1*. In hospitalized patients, two previously unrecognized loci were detected, namely, rs115545251 near *GFI1* and rs3181049 near *RAVER1*, while in general patients, rs11065822 near *CUX2* emerged as a newly identified locus. We also identified potential target genes, including those involved in inflammation signaling (*CARM1*), endothelial dysfunction (*INTS12*), and antiviral immune response (*RAVER1*), which may require further investigation.

**Conclusion:**

Our study offers insights into the genetic overlap between COVID-19 and immune factors, suggesting potential directions for future research and clinical exploration.

## 1 Introduction

COVID-19 is an extreme and clinically homogeneous disease phenotype (Gao et al., 2021; Oran and Topol, 2020). Infection with the coronavirus triggers the onset of the disease through a series of immune responses (Jackson et al., 2022). According to data from the World Health Organization (WHO), over 760 million cases and 6.9 million deaths have been recorded globally since December 2019, although the actual numbers are believed to be higher. The pandemic has not only disrupted global economic and trade development but has also imposed a significant socioeconomic burden on individuals, families, and societies worldwide. While most infected individuals experience mild to moderate respiratory illness and recover without specialized treatment, older adults or those with pre-existing conditions such as cardiovascular disease, diabetes, chronic respiratory diseases, or cancer are at a higher risk of severe outcomes or hospitalization. Notably, COVID-19 can affect individuals of any age and may lead to severe illness or death (Gao et al., 2021).

Epidemiological studies have identified severe risk factors associated with COVID-19, such as hypertension, coronary heart disease (Espiritu et al., 2024), type II diabetes (Hopkins et al., 2024), and thrombosis (Lindström et al., 2019). Furthermore, emerging evidence suggests that genetic factors play a significant role in determining susceptibility to COVID-19 (van der Made et al., 2020). To uncover the genetic basis of COVID-19, various genome-wide association studies (GWAS) have been conducted, with the largest study identifying 49 significant loci involving 24,202 severe cases (Pairo-Castineira et al., 2023). Recently, the development of GWAS multi-trait analysis (MTAG) provides a powerful approach to explore the genetic overlap and common etiologies between complex traits. By jointly analyzing multiple genetically correlated traits, MTAG can identify pleiotropic genetic variants that influence related phenotypes, revealing shared biological pathways and genetic interactions among various conditions.

Many studies have highlighted changes in blood measurements of COVID-19 patients (Zhou et al., 2021). COVID-19 is closely associated with various hematological abnormalities, including lymphopenia, elevated inflammatory markers such as C-reactive protein (CRP) and ferritin levels, as well as abnormalities in the coagulation pathway. These hematological changes provide important clues for understanding the mechanisms of the virus and its effects on the body. Notably, lymphopenia, although the underlying mechanisms remain unclear, has increasing evidence suggesting that lymphocytes play a crucial role in the pathophysiology of viral infections (Hegde, 2020; Song et al., 2024). Immune factors are closely related to blood cell counts, particularly white blood cell counts, indicating a strong relationship between the virus and the immune system, with immune factors playing significant roles in viral infections and disease progression (Jackson et al., 2022). Additionally, endothelial dysfunction is associated with COVID-19, and angiogenic T cells (Tangs) play a key role in endothelial repair (Liu et al., 2024). The emergence of a hypercoagulable state is also linked to COVID-19 (Valencia et al., 2024). Overall, this series of hematological risk factors interact to influence the course of COVID-19, with each factor potentially having a unique genetic background.

This study aims to quantify the genetic correlation between COVID-19 and related hematological traits. By conducting a comprehensive multi-trait genome-wide association study (MTAG), we identified common pleiotropic loci that influence both COVID-19 susceptibility and blood cell counts, providing deeper insights into the shared genetic architecture of COVID-19 and immune-related blood traits. These findings may help identify potential genetic markers that contribute to the clinical manifestations of COVID-19, thereby informing the development of targeted treatments.

## 2 Methods

### 2.1 GWAS data

The COVID-19 GWAS data used in this study are from the COVID-19 Host Genetics Initiative (HGI), specifically from the round seven release, using European population data. The study includes data on three different COVID-19 phenotypes: very severe respiratory confirmed COVID-19 vs. population (COVID-19-a2) with a total sample size of 1,072,442 and 13,769 cases; hospitalized COVID-19 vs. population (COVID-19-b2) with a total sample size of 2,062,805 and 32,519 cases; and general COVID-19 vs. population (COVID-19-c2) with a total sample size of 2,475,240 and 122,616 cases. The release date for this data is 8 April 2022. The study encompasses contributions from numerous research partners, including but not limited to Estonian Biobank (EstBB), UK Biobank (UKBB), FinnGen, Generation Scotland, deCODE, and the Million Veterans Program (MVP). Each dataset has been filtered and standardized to ensure consistency and reliability in the analysis. Specific details and descriptions of the datasets are provided in Supplementary Table S1.

For the study on hematological traits, data was sourced from the trans-ethnic and ancestry-specific blood-cell genetics study published in Cell. This comprehensive study investigated blood-cell genetics across 746,667 individuals from five global populations (Chen et al., 2020). For our research, we focused on the European subset, including data on lymphocyte count (524923 Europeans), basophil count (474,001 Europeans), eosinophil count (474237 Europeans), neutrophil count (519,288 Europeans), and monocyte count (521,594 Europeans). Specific details of the datasets are provided in Supplementary Table S2.

### 2.2 Global genetic correlation analysis

The cross-ancestry genetic correlation (Rg) between each pair of COVID-19 phenotypes and blood-cell traits was evaluated using LD Score Regression (LDSC) and GWAS summary statistics. LDSC is an established method that identifies genetic correlations between complex traits and diseases, providing etiological insights and helping to prioritize likely causal relationships. The primary challenges in estimating genetic correlation from GWAS data with traditional methods include the unavailability of individual genotype data and widespread sample overlap among meta-analyses. LDSC overcomes these challenges by using a technique called cross-trait LD Score regression, which requires only GWAS summary statistics and is not biased by sample overlap (Bulik-Sullivan et al., 2015). The formula used in LDSC is as follows:

$$E\left[\beta_{j}\gamma_{j}\right]=\frac{\sqrt{N_{1}N_{2}}r_{g}}{M}l+\frac{N_{s}r}{\sqrt{N_{1}N_{2}}}$$

where $\beta_{j}$ and $\gamma_{j\;}$ represent the effect sizes of ${SNP}_{j}$ on the two traits being tested, $N_{1}$ and $N_{2}$ are the sample sizes for the two traits, $N_{s}$ is the number of overlapping samples between the two traits, r is the phenotypic correlation in the overlapping samples, and, $l_{j}$ is the LD score. In this analysis, precomputed linkage disequilibrium scores for HapMap3 SNPs, derived from individuals of European ancestry in the 1000 Genomes Project (Bulik-Sullivan et al., 2015), were used. SNP markers with an imputation INFO score lower than 0.9 were excluded from the analysis (Bulik-Sullivan et al., 2015). This method allows for robust estimation of genetic correlations despite the complexities of sample overlap and lack of individual-level data.

### 2.3 Cell-type-specific enrichment of SNP heritability

Research has demonstrated that certain functional categories within the genome disproportionately contribute to the heritability of complex diseases (Trynka et al., 2013). Stratified LD Score Regression (s-LDSC) is a method developed to analyze these contributions by partitioning heritability from GWAS summary statistics while accounting for linkage disequilibrium (LD). This approach is computationally efficient and capable of handling large sample sizes, making it suitable for genome-wide studies involving extensive datasets. It leverages genome-wide information without relying solely on SNPs reaching genome-wide significance, allowing for a more comprehensive analysis. s-LDSC enables the identification of cell type-specific elements and other functional genomic regions that significantly contribute to the polygenic architecture of complex traits and diseases, providing insights into their biological mechanisms and helping prioritize genomic regions for further functional characterization (Finucane et al., 2015).

This study utilized annotation data constructed from six chromatin marks (DHS, H3K27ac, H3K36me3, H3K4me1, H3K4me3, and H3K9ac) across 88 cell types or tissues from the Roadmap project to partition the SNP heritability of various traits. For each chromatin mark, the cell type-specific annotations were further divided into nine categories: adipose, central nervous system, digestive system, cardiovascular, musculoskeletal and connective tissue, immune and blood, liver, pancreas, and others (Finucane et al., 2018). The annotation-specific enrichment values for each trait were converted into a color scale and visualized through hierarchical clustering.

### 2.4 Local genetic correlation analysis

To complement the genome-wide genetic correlation estimated by LDSC, which aggregates information across all variants in the genome, we employed ρ-HESS to quantify the local genetic correlation between pairs of traits (Finucane et al., 2018). ρ-HESS is designed to measure the correlation due to genetic variation at small regions in the genome, providing a more granular understanding of the genetic architecture. This technique only requires GWAS summary data and does not assume any specific distribution of causal variant effect sizes, while accounting for linkage disequilibrium (LD) and overlapping GWAS samples. The analysis using ρ-HESS involves several steps: first, it computes the eigenvalues of LD matrices and the squared projections of GWAS effect size vectors onto the eigenvectors of LD matrices for each trait. Next, it estimates the local SNP-heritability of each trait using these projections. Finally, ρ-HESS uses the local SNP-heritability estimates to calculate local genetic covariance estimates and their standard errors. In our study, all significant trait pairs identified in the global genetic correlation analysis (LDSC, *P* < 0.01) underwent local genetic correlation analysis using ρ-HESS. This allowed us to investigate which specific local genomic regions contributed to the global genetic correlation. A Bonferroni-corrected p-value of less than 0.05/n was considered statistically significant (n = Hypothesis testing quantity).

### 2.5 Multi-trait analysis of GWAS

Multi-Trait Analysis of GWAS (MTAG) is a method designed for the joint analysis of summary statistics from genome-wide association studies (GWAS) of different traits, possibly including overlapping samples. MTAG enhances the power to detect loci by analyzing GWAS summary statistics from related traits together, accounting for sample overlap and incomplete genetic correlation. It begins by filtering variants to remove non-common SNPs, duplicated SNPs, or SNPs with strand ambiguity (Turley et al., 2018). MTAG then estimates pairwise genetic correlations between traits using LD Score Regression (LDSC) and utilizes these estimates to calibrate the variance-covariance matrix of the random effect component (Bulik-Sullivan et al., 2015). Following this, MTAG performs a random-effect meta-analysis to produce SNP-level summary statistics. In our application, we prioritized nominally significant pleiotropic SNPs that achieved genome-wide significance (*P* < 1.67 × 10⁻⁸) in the multi-trait analysis and suggestive significance (*P* < 1 × 10^−3^) in the original single-trait GWAS. This threshold accounts for the number of independent tests conducted based on the traditional GWAS threshold of (*P* < 5 × 10⁻⁸), ensuring that the significance level is adjusted to minimize the risk of false positives. This method not only boosts the discovery of associated loci but also improves the interpretability of genetic associations through more informative bioinformatics analyses, thereby providing deeper insights into the genetic architecture of complex traits. In this study, our new loci refer to genetic loci that have not been reported in the relevant subtypes.

### 2.6 Colocalization and gene-based analysis

Colocalization (Giambartolomei et al., 2014) and gene-based analysis are critical for understanding the molecular basis of associations identified in genetic studies. This methodology integrates multiple association datasets, such as those from GWAS and gene expression studies, to assess whether two association signals are consistent with a shared causal variant. In our study, we applied this approach to investigate the relationship between COVID-19 and blood-cell traits. We used the Coloc tool to perform Bayesian colocalization analysis, aiming to identify loci where association signals for COVID-19 and blood-cell traits colocalize. This analysis uses default priors and considers a colocalization significant when the posterior probability of a shared causal variant (PP.H4) is greater than 0.8.

### 2.7 Transcriptome-wide association Study

To identify genes significantly associated with complex traits, we utilized S-PrediXcan with multiple eQTL datasets from GTEx v8 (a total of 49 tissues). The expression weights used were derived from GTEx v8 multi-tissue expression data, and were estimated and provided by Junghyun Jung from the Mancuso lab. In the S-PrediXcan analysis (Barbeira et al., 2018), we used gene expression and genetic variation (SNP) data from a small reference set of individuals to infer the expression of cis-acting genetic components in a larger set of phenotyped individuals based on their SNP genotype data. The predicted expression is presented in the form of a linear model, with weights determined by the correlation between SNPs and gene expression in the training data while accounting for linkage disequilibrium among SNPs. We associated gene expression with traits by conducting a transcriptome-wide association study (TWAS) to identify significant expression-trait associations. Based on Bonferroni correction, the genome-wide significance threshold for TWAS was set at *P*-TWAS <1 × 10^−6^ focusing on loci established to be associated with COVID-19 risk that also showed relevance in the TWAS analysis.

## 3 Results

### 3.1 Genetic correlations between COVID-19 and blood cell counts

**FIGURE 1:**
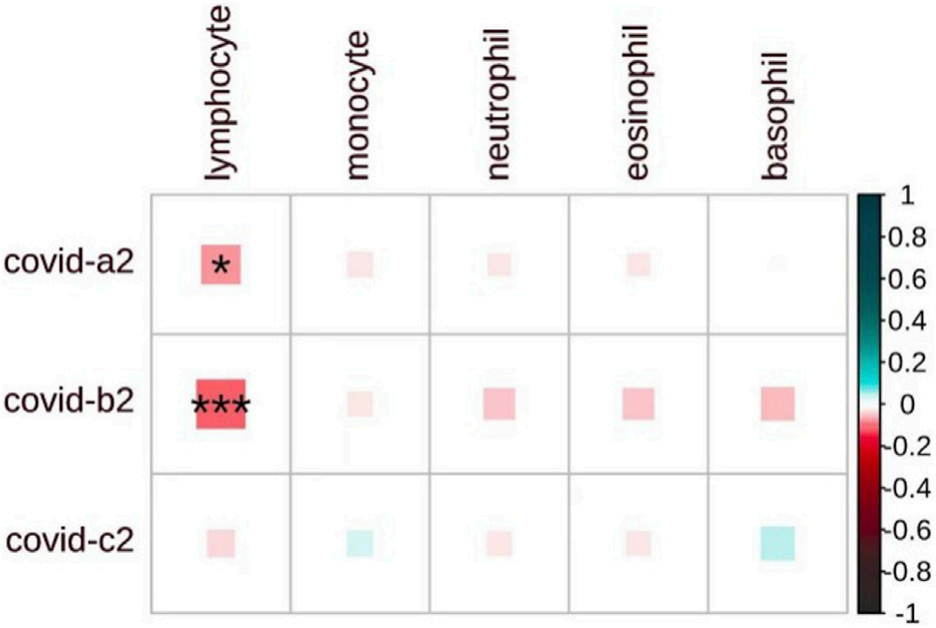
The heatmap displays the pairwise genome-wide genetic correlations between COVID-19 and blood cell counts. COVID-19-a2 for severe cases, COVID-19-b2 for hospitalized cases, and COVID-19-c2 for general cases. The matrix shows the strength of correlation (size of each square), with significant associations marked by an asterisk (P < 0.05). The shade of each square denotes a positive correlation (blue) or a negative correlation (red), with the depth of the shade reflecting the magnitude of the correlation.

We investigated the genetic correlation between COVID-19 and blood cell counts (Figure 1). Interestingly, while the correlation is weak among general patients (Rg = −0.03, p = 0.36), it becomes significantly stronger in severe patients (Rg = −0.07, p = 0.04) and hospitalized patients (Rg = −0.11, p = 2 × 10^−4^), suggesting a possible relationship with the severity of the disease. Importantly, our findings indicate a significant negative correlation between lymphocyte counts and COVID-19. Beyond lymphocytes, no significant correlations were found between COVID-19 and white blood cell counts (Supplementary Table S3). Most viral infections are associated with an increase in lymphocyte counts. Interestingly, several epidemiological studies have shown that COVID-19 infection leads to a decrease in lymphocyte levels (Chen et al., 2023; Niu et al., 2022; Xiong et al., 2020). Notably, this finding aligns with the results we obtained from our genetic analyses.

### 3.2 Cell type specific enrichment of SNP heritability

For pulmonary COVID-19 and its subtypes, we further divided the heritability of SNP according to six chromatin markers and nine cell types. We found that each subtype of COVID-19 patients showed a similar pattern in the six chromatin markers of lymphocyte count (Supplementary Figure S1). It is worth noting that the respiratory system tissues or cell types, such as lungs, significantly enrich the chromatin markers H3K27Ac, H3K4me3 and H3K4me1 of COVID-19 or its subtypes. We also observed significant genetic enrichment of COVID-19 and its subtypes in musculoskeletal/connective tissue, digestive system, nervous system and other tissue or cell types. Interestingly, we also observed significant genetic enrichment of T cells, NK cells, and monocytes related to immune factors in the blood system, consistent with previous research findings (Ma et al., 2021; Ma et al., 2022).

### 3.3 Local genetic correlation between COVID-19 and blood cell count

**FIGURE 2:**
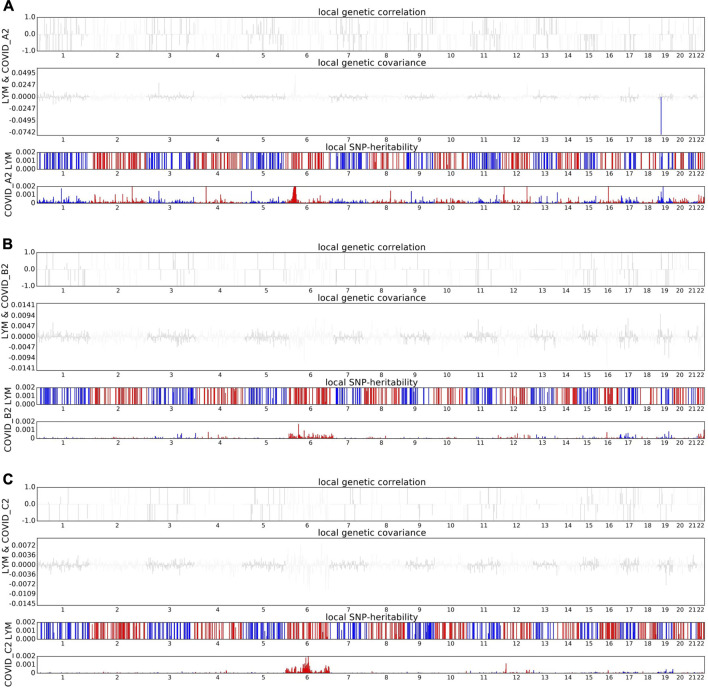
Local genetic correlation between COVID-19 and lymphocyte count. **(A)** The Manhattan plot shows the estimates of local genetic correlation, genetic covariance, and SNP heritability between severe COVID-19 and lymphocyte count. **(B)** The Manhattan plot shows the estimates of local genetic correlation, genetic covariance, and SNP heritability between hospitalized COVID-19 and lymphocyte count. **(C)** The Manhattan plot shows the estimates of local genetic correlation, genetic covariance, and SNP heritability between general COVID-19 and lymphocyte count. Red bars represent loci showing significant local genetic correlation after multiple testing adjustment (P < 0.05/sample size).

Building on these significant findings, we conducted a comprehensive whole genome scan to identify specific genomic regions that may influence the shared heritability of various genetically related traits (Supplementary Table S4). During our statistical analysis, we adjusted for multiple testing to ensure the accuracy and reliability of our results. Notably, we discovered a significant correlation between COVID-19 and lymphocyte counts, particularly in the specific genomic region 19p13.2. This finding is particularly noteworthy given that previous studies have indicated multiple GWAS signals in the 19p13.2 region associated with COVID-19 (Figure 2). This research not only reinforces the importance of the 19p13.2 locus in the genetic architecture of COVID-19 but also provides new insights and a deeper understanding of the genetic mechanisms underlying this disease (Ferreira et al., 2022).

### 3.4 Multi-trait analysis of GWAS

The evidence obtained from genetic correlation analysis has prompted us to further investigate the potential shared pleiotropic loci between COVID-19 and lymphocyte count. We employed Multi-Trait Analysis of GWAS (MTAG) to perform a multi-trait analysis of COVID-19 and its subtypes in relation to lymphocyte count, with special attention to SNPs achieving genome-wide significance (*P* < 1.66 × 10^−8^). To ensure the reliability of our results, we conducted comprehensive quality control (QC) analyses. The inflation lambda values for the original GWAS datasets were 1.055, 1.067, and 1.078 for severe COVID-19 (COVID-19-a2), hospitalized COVID-19 (COVID-19-b2), and general COVID-19 (COVID-19-c2), respectively, and 1.140 for lymphocyte count. The slightly higher lambda value for lymphocyte count may be attributed to its highly polygenic nature and the large sample size of the dataset, which can increase the observed inflation coefficient. In comparison, the MTAG results showed lower lambda values of 1.048 (COVID-19-a2), 1.041 (COVID-19-b2), and 1.023 (COVID-19-c2), indicating no significant inflation. Manhattan and QQ plots for these analyses are provided in Supplementary Figures S2, S3, confirming the robustness and consistency of our findings. These QC results further validate the reliability of our MTAG analysis and its ability to identify pleiotropic loci shared between COVID-19 and lymphocyte count.

In severe cases, we identified four loci, of which two are not reported previously. Notably, a signal at rs73009538 was discovered in the *CARM1* gene, which is implicated in regulating gene transcription, growth, and RNA splicing, among other biological processes (Lai et al., 2021). We also identified a locus (rs55779981) within the *RAVER1* gene region. Subsequently, among the four loci detected between hospitalized COVID-19 patients and lymphocyte count, two are not reported previously, specifically rs115545251 (1q22.1) and rs3181049 (19p13.3). The locus rs115545251 near the *GFI1* gene. It is worth noting that another locus associated with hospitalized patients (rs3181049) and a locus identified in severe patients (rs55779981) are both located in the *RAVER1* gene region. Interestingly, previous animal studies have suggested that other loci within the *RAVER1* region, such as rs74956615 (19q13.2), increase susceptibility to coronavirus through complex mechanisms (Fink-Baldauf et al., 2022). In other words, these three loci, including two newly identified and one known locus, are densely located within the *RAVER1* region yet remain independent of each other. For the general patient population, our LDSC analysis did not confirm a significant genetic correlation between COVID-19 and lymphocyte count. Despite the lack of significant genetic correlation, we conducted an MTAG analysis between hospitalized COVID-19 and lymphocyte counts, which revealed a previously unreported nominally significant loci (rs11065822) in the general population.

### 3.5 Colocalization

**FIGURE 3:**
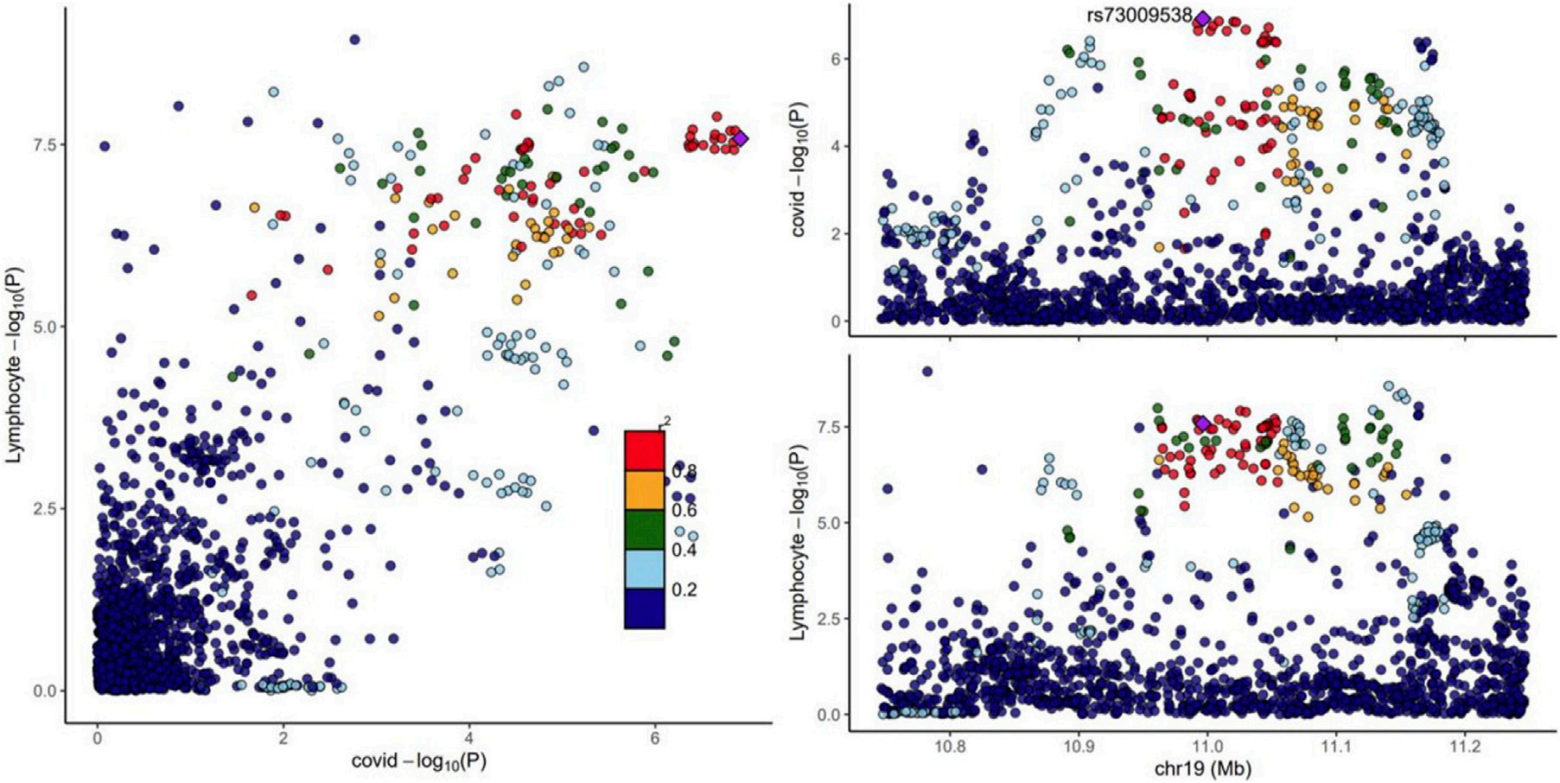
The regional plots of the 19p13.2 locus associated with COVID-19 using Locuscompare. New locus rs73009538. Purple diamond indicates the lead SNP, and circles represent the other SNPs in the region, with coloring from the linkage disequilibrium (r2, based on the 1000 Genomes Project Europeans) between each SNP and the lead SNP.

In this study, we employed the Coloc method to assess the Bayesian colocalization between COVID-19 and lymphocyte counts for each pleiotropic locus identified in the Multi-Trait Analysis of GWAS (MTAG). We identified three regions with PP.H4 values greater than 0.9, suggesting a high level of colocalization between COVID-19 and lymphocyte counts at these loci. Of the three colocalized loci, 19p13.2 was also identified in previous local genetic correlation analyses (Table1), reinforcing the consistency of our findings. This alignment not only bolsters the credibility of our genome-wide association results but also sheds new light on the genetic factors contributing to COVID-19 susceptibility, highlighting the potential for shared genetic pathways in immune response. To further explore the 19p13.2 (rs73009538) region in detail, we utilized the Locuscompare tool to visualize the association signals within this region. This analysis further confirmed the colocalization of genetic signals for COVID-19 susceptibility and lymphocyte counts at 19q13.2 (Figure 3) (Pruim et al., 2010).

**TABLE 1:** Multi-trait meta-analysis between COVID-19 and lymphocyte.

Type of COVID-19	SNP	CHR	BP	A1	A2	Trait 1		Trait 2		MTAG		Genes	Annotation	PP.H4 (Coloc)
						Beta	*P*-value	Beta	*P*-value	Beta	*P*-mtag			
Severe	rs7515509	1	77949123	G	A	0.0799	7.50 × 10^−8^	0.0096	6.57 × 10^−7^	1.8816	1.16 × 10^−8^	*ZZZ3*	known	1.00
	rs10059611	5	131787278	G	T	0.0767	2.24 × 10^−7^	0.0195	1.27 × 10^−21^	1.9687	5.08 × 10^−9^	*IRF1*	known	0.02
	rs55779981	19	10437764	T	C	−0.0256	1.20 × 10^−6^	−0.0318	2.76 × 10^−35^	−2.3831	9.46 × 10^−9^	*RAVER1*	new	0.02
	rs73009538	19	10996391	C	T	0.0832	1.22 × 10^−7^	0.0120	2.63 × 10^−8^	2.0260	1.55 × 10^−8^	*CARM1*	new	0.94
Hospitalized	rs115545251	1	93042753	G	A	0.1458	2.10 × 10^−7^	0.0347	2.23 × 10^−11^	4.4961	5.08 × 10^−9^	*GFI1*	new	1.00
	rs7664615	4	25448493	A	G	−0.0726	6.18 × 10^−8^	−0.0080	1.46 × 10^−3^	−1.8722	1.58 × 10^−8^	*ANAPC4*	known	0.07
	rs72669986	4	106550272	C	A	0.0915	2.33 × 10^−7^	0.0189	2.26 × 10^−7^	2.8609	1.79 × 10^−8^	*ARHGEF38*	known	0.03
	rs7254272	19	4069119	G	A	0.0657	1.16 × 10^−7^	0.0123	4.03 × 10^−4^	−1.9515	2.35 × 10^−8^	*ZBTB7A*	known	0.00
	rs3181049	19	10441117	G	A	−0.0654	9.06 × 10^−8^	−0.0318	3.45 × 10^−35^	−2.1767	3.03 × 10^−11^	*RAVER1*	new	0.02
General	rs11065822	12	111600134	G	T	−0.0207	6.46 × 10^−5^	0.0585	6.90 × 10^−180^	−1.4317	4.01 × 10^−11^	*CUX2*	new	0.16

PP.H4, posterior probability of shared causal variant.

### 3.6 Transcriptome-wide association study (TWAS) and gene analysis

We conducted a Transcriptome-Wide Association Study (TWAS) to investigate gene expression profiles associated with COVID-19 susceptibility and related phenotypes. TWAS integrates genotype and expression data, allowing for the identification of genes significantly linked to specific traits, thereby offering insights into gene regulation and disease association. Additionally, we have included Manhattan plots and QQ plots for the TWAS analyses, which can be accessed on Figshare (DOI: 10.6084/m9.figshare.28005845). In most tissues, the TWAS inflation factor (λ > 1.2) indicates moderate inflation, likely driven by the large sample size of the COVID-19 dataset. Similar levels of inflation have been observed in Alzheimer’s disease studies (Wingo et al., 2021), reflecting the increased statistical power to detect polygenic associations in large-scale datasets. These findings suggest that the observed inflation is primarily attributable to true signals rather than systematic bias.

The findings were largely consistent with earlier studies (Pathak et al., 2020) (Supplementary Tables S5–S7). In severe case A2, the eQTL rs10059611 was associated with *ACSL6* in brain tissue, LncRNA in leukocytes and the stomach, microRNA in lung, and *RAD50* in the uterus, pancreas, and skeletal muscle. These associations suggest that rs10059611 might have pleiotropic effects across multiple tissues, influencing diverse biological pathways relevant to severe COVID-19 outcomes. In hospitalized case B2, the eQTL rs7664615 was associated with *PI4K2B* in the pituitary, a gene involved in early T cell activation and immune regulation. In the thyroid, it was linked to *SLC34A2,* which plays a role in phosphate transport and cellular metabolism, and in the cerebellum, it was connected to *ZCCHC4*, a zinc finger protein involved in RNA processing and regulation. Additionally, rs72669986, located in the *ARHGEF38* gene, was associated with *GSTCD* in the breast, esophagus, skin, and tibial nerve; *SLC34A2* in the thyroid; and *INTS12* in leukocytes, fibroblasts, prostate, and thyroid.

Furthermore, the TWAS analysis identified loci that provide additional insights into discoveries from GWAS studies. In severe case A2, the locus rs2236645 on chromosome 21 was associated with *ATP5PO* in the lung, brain, and skin, as well as *MRPS6* in the esophagus and left atrium. Both genes are involved in mitochondrial function, a pathway that may play a critical role during early COVID-19 infection, as the virus relies on enhanced mitochondrial metabolism to support replication. In hospitalized case B2, rs3785632 was linked to *SNHG26*, a long non-coding RNA (lncRNA), and rs717624 was associated with *NCOR1* in the testis, which regulates transcriptional activity. Additionally, rs3910266 was connected to *SNX19* in the brain, a gene potentially involved in intracellular trafficking and cellular response mechanisms.

## 4 Discussion

This study is one of the first comprehensive investigations into the genetic underpinnings of severe COVID-19, specifically focusing on the interplay between inflammation-induced lung damage and genetic susceptibility. It is evident that severe cases are largely driven by inflammation-mediated by immunological factors, with the progression and severity of the disease influenced by both environmental and genetic factors. Our findings highlight the crucial role of genetic determinants in shaping susceptibility, disease progression, and mortality in COVID-19, underscoring the need for further research to better understand these mechanisms and inform the development of targeted therapeutic interventions.

Recently, a Genome-Wide Association Study (GWAS) for COVID-19, launched by the GenOMICC, ISARIC4C, and SCOURGE consortia, collected genetic data from 24,202 severe COVID-19 patients across diverse ancestries. This study identified 49 genome-wide significant associations, of which 16 were newly reported (Pairo-Castineira et al., 2023). Beyond the known features of COVID-19 immune susceptibility, we found that several established COVID-19 genetic loci also impact the inflammatory response, a key risk factor for the progression and prognosis of COVID-19.

Based on the aforementioned evidence, we investigated the shared genetic architecture between different COVID-19 subtypes and other leukocytes. We observed a widespread negative correlation between lymphocyte counts and COVID-19, with a stronger correlation and more pronounced reduction in lymphocyte counts observed in more severe cases, such as critical and hospitalized patients. This phenomenon can be attributed to the direct viral infection of lymphocytes, particularly T cells and B cells, resulting in cellular death and a reduction in lymphocyte numbers (Rouse and Horohov, 1986). Concurrently, the immune system initiates a robust inflammatory response, which may escalate into a cytokine storm. The massive release of inflammatory mediators can induce lymphocyte apoptosis (programmed cell death), further diminishing lymphocyte numbers (Delogu et al., 2008; Peppelenbosch and van Deventer, 2004). Additionally, COVID-19 infection may cause immune suppression (Abbasi, 2021; Mehta et al., 2020), especially in severe cases. The increase in suppressive cytokines, such as IL-10 and TGF-β, may inhibit lymphocyte proliferation and function, further reducing their numbers. In summary, lymphopenia induced by COVID-19 infection is a complex process potentially involving direct viral effects, immune response, and immune suppression, with these pathophysiological responses being more pronounced in severe cases compared to milder ones. This suggests that lymphocyte count may partially reflect disease severity and prognosis. This finding can complement widely used imaging techniques, aiding clinicians in more effectively assessing patient conditions and guiding the design of clinical treatment plans. By monitoring lymphocyte counts, we can more accurately track disease progression and adjust therapeutic strategies as needed, ultimately improving overall patient outcomes.

Given the strong association between viral infection and lymphocyte count, we conducted deeper analyses to explore the shared genetic mechanisms between these phenotypes. We identified significant local genetic associations in the 19p13.2 region among severely ill patients, supported by previous studies (Ferreira et al., 2022). To deepen our analysis, we employed the MTAG approach to identify shared genetic loci between COVID-19 subtypes and lymphocyte count. By enhancing statistical power, we successfully identified 14 pleiotropic loci associated with COVID-19 and lymphocyte count, five of which were newly discovered and previously unreported in this subtype.

In severe cases, four nominally significant loci were identified, two of which are not reported previously. rs73009538 in the *CARM1* region associated with type I interferon, and the deficiency of type I IFN response is one of the key factors in severe COVID-19 (Hadjadj et al., 2020). *CARM1* is crucial for lung epithelial cell function (O'Brien et al., 2010), and its deficiency can lead to respiratory developmental issues, possibly linked to its role in gene transcription and cell growth. Additionally, *CARM1* acts as a transcriptional coactivator for NF-kappaB, significantly influencing inflammatory responses (Srour et al., 2022). Notably, research from the Getx study found that rs73009538 serves as an eQTL for *SMARCA4*, a key component of the SWI/SNF complex that regulates immune responses and gene expression (Rhoads et al., 2018), particularly ACE2, the primary receptor for COVID-19 (Zoufaly et al., 2020). Mutations in *SMARCA4* may disrupt mSWI/SNF complex function, resulting in lower ACE2 levels and potentially reducing cellular susceptibility to viral infection (Wei et al., 2023). *RAVER1* is a key factor in inducing antiviral responses, capable of promoting the host’s immune reaction to viruses and enhancing antiviral capabilities (Chen et al., 2013). Meanwhile, *RAVER1* is involved in mediating inflammation-related cell death processes, thereby regulating apoptosis or other pathways of cell death (Malireddi et al., 2023; Zhang et al., 2024). A deficiency in *RAVER1* may lead to increased susceptibility of the host to COVID-19, potentially resulting in a more severe progression of the disease following infection. Interestingly, rs10059611 is an eQTL of LINC02863. While lincRNAs usually do not encode proteins, some small ORFs within them can be translated, though the mechanisms are unclear (Matsumoto et al., 2017). Additionally, lincRNAs can regulate protein-coding gene expression by interacting with RNA, DNA, or proteins (Atianand et al., 2017; Kong et al., 2024), influencing immune cell development (Brazão et al., 2016) and inflammation suppression (Atianand et al., 2016; Yue et al., 2020).

We identified five nominally significant loci associated with hospitalized COVID-19 cases, including two previously unreported loci: rs115545251 (located at 1q22.1), rs3181049 (located at 19q13.3). The rs115545251 locus, located near the *GFI1* gene, is linked to hospitalized COVID-19 cases, with colocalization analysis confirming its association with lymphocyte count. The *GFI1* gene, which encodes a zinc finger protein, promotes the proliferation of lymphocytes and granulocytes and functions as a transcriptional repressor involved in various biological activities. Mutations in the *GFI1* gene, which is crucial for lymphocyte proliferation and regulation (Guo et al., 2021; Möröy and Khandanpour, 2011; 2019),can lead to lymphopenia and enhanced Th2 inflammatory responses (Sarkar et al., 2021; Zhu et al., 2006), potentially resulting in immune dysregulation and severe COVID-19 progression, highlighting the importance of rs115545251 in this context. The locus identified in hospitalized patients (rs3181049) and the locus found in severe patients (rs55779981) are both located in the *RAVER1* gene region, further supporting our hypothesis: The defects in the *RAVER1* gene not only reduce antiviral capability but also increase susceptibility to the disease, thereby raising the incidence of severe cases.

Additionally, our transcriptome-wide association study identified the rs72669986 locus in the *ARHGEF38* region as a potential regulator of *INTS12* expression, which is predominantly found in epithelial and cells and plays a critical role in cellular activity by regulating protein synthesis pathways (Kheirallah et al., 2017; Obeidat et al., 2013). *INTS12* may influence cellular signaling pathways essential for the proliferation, differentiation, and survival of cells, thereby impacting COVID-19 susceptibility and disease severity while interacting with various genes related to lung function within complex gene networks (Obeidat et al., 2013). These findings shed light on the molecular mechanisms governing lung function and suggest potential targets for future interventions and treatments for related diseases. The eQTL rs7664615, located on *ANAPC4* in GWAS. In the pituitary, it projects to the *PI4K2B* gene, whose encoded protein is involved in early T cell activation; in the thyroid, it projects to *SLC34A2*, which codes for a protein that regulates transmembrane protein transport; and in the cerebellum, it projects to *ZCCHC4*. COVID-19 can directly or indirectly affect the thyroid, leading to thyroid-related diseases, possibly secondary to the hypothalamic-pituitary-thyroid (HPT) axis, which may be related to the virus’s impact on the immune system (Lui et al., 2024). Additionally, thyroid hormones play a critical role as regulators of immune activity at the cellular level (Jaeger et al., 2021), contributing to lymphocyte homeostasis (De Vito et al., 2011). The zinc finger protein encoded by *ZCCHC4* is involved in the process of viral clearance and plays an important role in regulating both innate and adaptive immune responses (Fu and Blackshear, 2017).

While our study provides valuable insights into the shared genetic architecture between COVID-19 and immune-related traits, several limitations should be noted. First, the lack of integration with single-cell RNA sequencing (scRNA-seq) data limits the resolution of our findings, particularly in identifying specific immune cell subtypes and pathways. Future research could leverage methods like scPagwas (Ma et al., 2023) to explore these aspects further. Additionally, functional validation of the identified loci was beyond the scope of this study, leaving the mechanistic roles of these loci to be explored in future experiments. Another key limitation is the use of binary and quantitative traits in our MTAG analysis. While this method may present some challenges in terms of accuracy and interpretability, it has been widely used in similar studies and remains a reasonable approach under the current constraints (Chang et al., 2024; Guo et al., 2020; Jiang et al., 2024). Lastly, the study primarily focuses on European ancestry populations, and further research is needed to assess the generalizability of these findings across diverse ancestries.

In this study, we conducted an in-depth multi-phenotype analysis of various COVID-19 subtypes and lymphocyte counts. By jointly analyzing these traits and enhancing statistical power, we identified five not reported previously genetic loci, shedding light on the pleiotropic genetic architecture between them. While some loci may exhibit vertical pleiotropy due to the causal relationship between COVID-19 and lymphocyte counts, others may display horizontal pleiotropy, directly influencing both traits. Further functional studies are needed to investigate these loci and their underlying mechanisms, contributing to a deeper understanding of COVID-19 susceptibility.

## Data Availability

The original contributions presented in the study are included in the article/Supplementary Material, further inquiries can be directed to the corresponding authors.

## References

[B1] Abbasi J. (2021). Researchers tie severe immunosuppression to chronic COVID-19 and virus variants. Jama 325 (20), 2033–2035. 10.1001/jama.2021.7212 33950236

[B2] Atianand M. K. Caffrey D. R. Fitzgerald K. A. (2017). Immunobiology of long noncoding RNAs. Annu. Rev. Immunol. 35, 177–198. 10.1146/annurev-immunol-041015-055459 28125358 PMC6449690

[B3] Atianand M. K. Hu W. Satpathy A. T. Shen Y. Ricci E. P. Alvarez-Dominguez J. R. (2016). A long noncoding RNA lincRNA-EPS acts as a transcriptional brake to restrain inflammation. Cell 165 (7), 1672–1685. 10.1016/j.cell.2016.05.075 27315481 PMC5289747

[B4] Barbeira A. N. Dickinson S. P. Bonazzola R. Zheng J. Wheeler H. E. Torres J. M. (2018). Exploring the phenotypic consequences of tissue specific gene expression variation inferred from GWAS summary statistics. Nat. Commun. 9 (1), 1825. 10.1038/s41467-018-03621-1 29739930 PMC5940825

[B5] Brazão T. F. Johnson J. S. Müller J. Heger A. Ponting C. P. Tybulewicz V. L. (2016). Long noncoding RNAs in B-cell development and activation. Blood 128 (7), e10–e19. 10.1182/blood-2015-11-680843 27381906 PMC5000579

[B6] Bulik-Sullivan B. Finucane H. K. Anttila V. Gusev A. Day F. R. Loh P. R. (2015). An atlas of genetic correlations across human diseases and traits. Nat. Genet. 47 (11), 1236–1241. 10.1038/ng.3406 26414676 PMC4797329

[B7] Chang X. Zhi L. Jiang Y. Yu L. Li L. Song Y. (2024). Multi-trait genetic analysis of asthma and eosinophils uncovers novel loci in east asians. 10.21203/rs.3.rs-5425540/v1 PMC12126482 40450010

[B8] Chen G. Zhao X. Chen X. Liu C. (2023). Early decrease in blood lymphocyte count is associated with poor prognosis in COVID-19 patients: a retrospective cohort study. BMC Pulm. Med. 23 (1), 453. 10.1186/s12890-023-02767-z 37986163 PMC10662697

[B9] Chen H. Li Y. Zhang J. Ran Y. Wei J. Yang Y. (2013). RAVER1 is a coactivator of MDA5-mediated cellular antiviral response. J. Mol. Cell Biol. 5 (2), 111–119. 10.1093/jmcb/mjt006 23390309

[B10] Chen M. H. Raffield L. M. Mousas A. Sakaue S. Huffman J. E. Moscati A. (2020). Trans-ethnic and ancestry-specific blood-cell genetics in 746,667 individuals from 5 global populations. Cell 182 (5), 1198–1213.e14. 10.1016/j.cell.2020.06.045 32888493 PMC7480402

[B11] Delogu G. Famularo G. Tellan G. Marandola M. Antonucci A. Signore M. (2008). Lymphocyte apoptosis, caspase activation and inflammatory response in septic shock. Infection 36 (5), 485–487. 10.1007/s15010-008-7070-y 18791840

[B12] De Vito P. Incerpi S. Pedersen J. Z. Luly P. Davis F. B. Davis P. J. (2011). Thyroid hormones as modulators of immune activities at the cellular level. Thyroid 21 (8), 879–890. 10.1089/thy.2010.0429 21745103

[B13] Espiritu A. I. Pilapil J. C. A. Aherrera J. A. M. Sy M. C. C. Anlacan V. M. M. Villanueva E. Q. I. (2024). Outcomes of patients with COVID-19 and coronary artery disease and heart failure: findings from the Philippine CORONA study. BMC Res. Notes 17 (1), 14. 10.1186/s13104-023-06677-5 38178236 PMC10768280

[B14] Ferreira L. C. Gomes C. E. M. Rodrigues-Neto J. F. Jeronimo S. M. B. (2022). Genome-wide association studies of COVID-19: connecting the dots. Infect. Genet. Evol. 106, 105379. 10.1016/j.meegid.2022.105379 36280088 PMC9584840

[B15] Fink-Baldauf I. M. Stuart W. D. Brewington J. J. Guo M. Maeda Y. (2022). CRISPRi links COVID-19 GWAS loci to LZTFL1 and RAVER1. EBioMedicine 75, 103806. 10.1016/j.ebiom.2021.103806 34998241 PMC8731227

[B16] Finucane H. K. Bulik-Sullivan B. Gusev A. Trynka G. Reshef Y. Loh P. R. (2015). Partitioning heritability by functional annotation using genome-wide association summary statistics. Nat. Genet. 47 (11), 1228–1235. 10.1038/ng.3404 26414678 PMC4626285

[B17] Finucane H. K. Reshef Y. A. Anttila V. Slowikowski K. Gusev A. Byrnes A. (2018). Heritability enrichment of specifically expressed genes identifies disease-relevant tissues and cell types. Nat. Genet. 50 (4), 621–629. 10.1038/s41588-018-0081-4 29632380 PMC5896795

[B18] Fu M. Blackshear P. J. (2017). RNA-binding proteins in immune regulation: a focus on CCCH zinc finger proteins. Nat. Rev. Immunol. 17 (2), 130–143. 10.1038/nri.2016.129 27990022 PMC5556700

[B19] Gao M. Piernas C. Astbury N. M. Hippisley-Cox J. O'Rahilly S. Aveyard P. (2021). Associations between body-mass index and COVID-19 severity in 6·9 million people in England: a prospective, community-based, cohort study. Lancet Diabetes Endocrinol. 9 (6), 350–359. 10.1016/s2213-8587(21)00089-9 33932335 PMC8081400

[B20] Giambartolomei C. Vukcevic D. Schadt E. E. Franke L. Hingorani A. D. Wallace C. (2014). Bayesian test for colocalisation between pairs of genetic association studies using summary statistics. PLoS Genet. 10 (5), e1004383. 10.1371/journal.pgen.1004383 24830394 PMC4022491

[B21] Guo Y. Rist P. M. Daghlas I. Giulianini F. Kurth T. Chasman D. I. (2020). A genome-wide cross-phenotype meta-analysis of the association of blood pressure with migraine. Nat. Commun. 11 (1), 3368. 10.1038/s41467-020-17002-0 32632093 PMC7338361

[B22] Guo Z. Zhang Z. Prajapati M. Li Y. (2021). Lymphopenia caused by virus infections and the mechanisms beyond. Viruses 13 (9), 1876. 10.3390/v13091876 34578457 PMC8473169

[B23] Hadjadj J. Yatim N. Barnabei L. Corneau A. Boussier J. Smith N. (2020). Impaired type I interferon activity and inflammatory responses in severe COVID-19 patients. Science 369 (6504), 718–724. 10.1126/science.abc6027 32661059 PMC7402632

[B24] Hegde S. (2020). Many paths to COVID-19 lymphocyte dysfunction. Nat. Rev. Immunol. 20 (7), 408. 10.1038/s41577-020-0361-y PMC7273380 32504059

[B25] Hopkins R. Young K. G. Thomas N. J. Godwin J. Raja D. Mateen B. A. (2024). Risk factor associations for severe COVID-19, influenza and pneumonia in people with diabetes to inform future pandemic preparations: UK population-based cohort study. BMJ Open 14 (1), e078135. 10.1136/bmjopen-2023-078135 PMC10831438 38296292

[B26] Jackson C. B. Farzan M. Chen B. Choe H. (2022). Mechanisms of SARS-CoV-2 entry into cells. Nat. Rev. Mol. Cell Biol. 23 (1), 3–20. 10.1038/s41580-021-00418-x 34611326 PMC8491763

[B27] Jaeger M. Sloot Y. J. E. Horst R. T. Chu X. Koenen H. Koeken V. (2021). Thyrotrophin and thyroxine support immune homeostasis in humans. Immunology 163 (2), 155–168. 10.1111/imm.13306 33454989 PMC8114202

[B28] Jiang Y. Song Y. Li Y. Tong Y. Ding H. Li L. (2024). Multi-trait genetic analysis identifies novel pleiotropic loci for stroke and hematological traits or risk factors. Fundam. Res. 10.1016/j.fmre.2024.05.004 PMC13424371 42539752

[B29] Kheirallah A. K. de Moor C. H. Faiz A. Sayers I. Hall I. P. (2017). Lung function associated gene Integrator Complex subunit 12 regulates protein synthesis pathways. BMC Genomics 18 (1), 248. 10.1186/s12864-017-3628-3 28335732 PMC5364626

[B30] Kong X. Wang Q. Wang X. Yang K. Nie S. Li Y. (2024). LINC01002 functions as a ceRNA to regulate FRMD8 by sponging miR-4324 for the development of COVID-19. Virol. J. 21 (1), 109. 10.1186/s12985-024-02382-2 38734674 PMC11088083

[B31] Lai Y. Li X. Li T. Nyunoya T. Chen K. Kitsios G. D. (2021). Endotoxin stabilizes protein arginine methyltransferase 4 (PRMT4) protein triggering death of lung epithelia. Cell Death Dis. 12 (9), 828. 10.1038/s41419-021-04115-7 34480022 PMC8414963

[B32] Lindström S. Wang L. Smith E. N. Gordon W. van Hylckama Vlieg A. de Andrade M. (2019). Genomic and transcriptomic association studies identify 16 novel susceptibility loci for venous thromboembolism. Blood 134 (19), 1645–1657. 10.1182/blood.2019000435 31420334 PMC6871304

[B33] Liu X. Hua L. Chu J. Zhou W. Jiang F. Wang L. (2024). Endothelial dysfunction and disease severity in COVID-19: insights from circulating Tang cell counts as a potential biomarker. Int. Immunopharmacol. 130, 111788. 10.1016/j.intimp.2024.111788 38447419

[B34] Lui D. T. W. Lee C. H. Woo Y. C. Hung I. F. N. Lam K. S. L. (2024). Thyroid dysfunction in COVID-19. Nat. Rev. Endocrinol. 20 (6), 336–348. 10.1038/s41574-023-00946-w 38347167

[B35] Ma Y. Deng C. Zhou Y. Zhang Y. Qiu F. Jiang D. (2023). Polygenic regression uncovers trait-relevant cellular contexts through pathway activation transformation of single-cell RNA sequencing data. Cell Genom 3 (9), 100383. 10.1016/j.xgen.2023.100383 37719150 PMC10504677

[B36] Ma Y. Huang Y. Zhao S. Yao Y. Zhang Y. Qu J. (2021). Integrative genomics analysis reveals a 21q22.11 locus contributing risk to COVID-19. Hum. Mol. Genet. 30 (13), 1247–1258. 10.1093/hmg/ddab125 33949668 PMC8136003

[B37] Ma Y. Qiu F. Deng C. Li J. Huang Y. Wu Z. (2022). Integrating single-cell sequencing data with GWAS summary statistics reveals CD16+monocytes and memory CD8+T cells involved in severe COVID-19. Genome Med. 14 (1), 16. 10.1186/s13073-022-01021-1 35172892 PMC8851814

[B38] Malireddi R. K. S. Bynigeri R. R. Mall R. Nadendla E. K. Connelly J. P. Pruett-Miller S. M. (2023). Whole-genome CRISPR screen identifies RAVER1 as a key regulator of RIPK1-mediated inflammatory cell death, PANoptosis. iScience 26 (6), 106938. 10.1016/j.isci.2023.106938 37324531 PMC10265528

[B39] Matsumoto A. Pasut A. Matsumoto M. Yamashita R. Fung J. Monteleone E. (2017). mTORC1 and muscle regeneration are regulated by the LINC00961-encoded SPAR polypeptide. Nature 541 (7636), 228–232. 10.1038/nature21034 28024296

[B40] Mehta P. McAuley D. F. Brown M. Sanchez E. Tattersall R. S. Manson J. J. (2020). COVID-19: consider cytokine storm syndromes and immunosuppression. Lancet 395 (10229), 1033–1034. 10.1016/s0140-6736(20)30628-0 32192578 PMC7270045

[B41] Möröy T. Khandanpour C. (2011). Growth factor independence 1 (Gfi1) as a regulator of lymphocyte development and activation. Semin. Immunol. 23 (5), 368–378. 10.1016/j.smim.2011.08.006 21920773

[B42] Möröy T. Khandanpour C. (2019). Role of GFI1 in epigenetic regulation of MDS and AML pathogenesis: mechanisms and therapeutic implications. Front. Oncol. 9, 824. 10.3389/fonc.2019.00824 31508375 PMC6718700

[B43] Niu J. Sareli C. Mayer D. Visbal A. Sareli A. (2022). Lymphopenia as a predictor for adverse clinical outcomes in hospitalized patients with COVID-19: a single center retrospective study of 4485 cases. J. Clin. Med. 11 (3), 700. 10.3390/jcm11030700 35160150 PMC8837002

[B44] Obeidat M. Miller S. Probert K. Billington C. K. Henry A. P. Hodge E. (2013). GSTCD and INTS12 regulation and expression in the human lung. PLoS One 8 (9), e74630. 10.1371/journal.pone.0074630 24058608 PMC3776747

[B45] O'Brien K. B. Alberich-Jordà M. Yadav N. Kocher O. Diruscio A. Ebralidze A. (2010). CARM1 is required for proper control of proliferation and differentiation of pulmonary epithelial cells. Development 137 (13), 2147–2156. 10.1242/dev.037150 20530543 PMC2882134

[B46] Oran D. P. Topol E. J. (2020). Prevalence of asymptomatic SARS-CoV-2 infection: a narrative review. Ann. Intern Med. 173 (5), 362–367. 10.7326/m20-3012 32491919 PMC7281624

[B47] Pairo-Castineira E. Rawlik K. Bretherick A. D. Qi T. Wu Y. Nassiri I. (2023). GWAS and meta-analysis identifies 49 genetic variants underlying critical COVID-19. Nature 617 (7962), 764–768. 10.1038/s41586-023-06034-3 37198478 PMC10208981

[B48] Pathak G. A. Singh K. Miller-Fleming T. W. Wendt F. R. Ehsan N. Hou K. (2020). Integrative analyses identify susceptibility genes underlying COVID-19 hospitalization. medRxiv. 10.1101/2020.12.07.20245308 PMC8316582 34315903

[B49] Peppelenbosch M. P. van Deventer S. J. (2004). T cell apoptosis and inflammatory bowel disease. Gut 53 (11), 1556–1558. 10.1136/gut.2004.040824 15479669 PMC1774279

[B50] Pruim R. J. Welch R. P. Sanna S. Teslovich T. M. Chines P. S. Gliedt T. P. (2010). LocusZoom: regional visualization of genome-wide association scan results. Bioinformatics 26 (18), 2336–2337. 10.1093/bioinformatics/btq419 20634204 PMC2935401

[B51] Rhoads T. W. Burhans M. S. Chen V. B. Hutchins P. D. Rush M. J. P. Clark J. P. (2018). Caloric restriction engages hepatic RNA processing mechanisms in rhesus monkeys. Cell Metab. 27 (3), 677–688. 10.1016/j.cmet.2018.01.014 29514073 PMC5844481

[B52] Rouse B. T. Horohov D. W. (1986). Immunosuppression in viral infections. Rev. Infect. Dis. 8 (6), 850–873. 10.1093/clinids/8.6.850 3025993 PMC7792945

[B53] Sarkar M. H. Yagi R. Endo Y. Koyama-Nasu R. Wang Y. Hasegawa I. (2021). IFNγ suppresses the expression of GFI1 and thereby inhibits Th2 cell proliferation. PLoS One 16 (11), e0260204. 10.1371/journal.pone.0260204 34807911 PMC8608330

[B54] Song M. Liu X. Shen W. Wang Z. Wu J. Jiang J. (2024). IFN-γ decreases PD-1 in T lymphocytes from convalescent COVID-19 patients via the AKT/GSK3β signaling pathway. Sci. Rep. 14 (1), 5038. 10.1038/s41598-024-55191-6 38424104 PMC10904811

[B55] Srour N. Khan S. Richard S. (2022). The influence of arginine methylation in immunity and inflammation. J. Inflamm. Res. 15, 2939–2958. 10.2147/jir.S364190 35602664 PMC9114649

[B56] Trynka G. Sandor C. Han B. Xu H. Stranger B. E. Liu X. S. (2013). Chromatin marks identify critical cell types for fine mapping complex trait variants. Nat. Genet. 45 (2), 124–130. 10.1038/ng.2504 23263488 PMC3826950

[B57] Turley P. Walters R. K. Maghzian O. Okbay A. Lee J. J. Fontana M. A. (2018). Multi-trait analysis of genome-wide association summary statistics using MTAG. Nat. Genet. 50 (2), 229–237. 10.1038/s41588-017-0009-4 29292387 PMC5805593

[B58] Valencia I. Lumpuy-Castillo J. Magalhaes G. Sánchez-Ferrer C. F. Lorenzo Ó. Peiró C. (2024). Mechanisms of endothelial activation, hypercoagulation and thrombosis in COVID-19: a link with diabetes mellitus. Cardiovasc Diabetol. 23 (1), 75. 10.1186/s12933-023-02097-8 38378550 PMC10880237

[B59] van der Made C. I. Simons A. Schuurs-Hoeijmakers J. van den Heuvel G. Mantere T. Kersten S. (2020). Presence of genetic variants among young men with severe COVID-19. Jama 324 (7), 663–673. 10.1001/jama.2020.13719 32706371 PMC7382021

[B60] Wei J. Patil A. Collings C. K. Alfajaro M. M. Liang Y. Cai W. L. (2023). Pharmacological disruption of mSWI/SNF complex activity restricts SARS-CoV-2 infection. Nat. Genet. 55 (3), 471–483. 10.1038/s41588-023-01307-z 36894709 PMC10011139

[B61] Wingo A. P. Liu Y. Gerasimov E. S. Gockley J. Logsdon B. A. Duong D. M. (2021). Integrating human brain proteomes with genome-wide association data implicates new proteins in Alzheimer's disease pathogenesis. Nat. Genet. 53 (2), 143–146. 10.1038/s41588-020-00773-z 33510477 PMC8130821

[B62] Xiong S. Liu L. Lin F. Shi J. Han L. Liu H. (2020). Clinical characteristics of 116 hospitalized patients with COVID-19 in Wuhan, China: a single-centered, retrospective, observational study. BMC Infect. Dis. 20 (1), 787. 10.1186/s12879-020-05452-2 33092539 PMC7578439

[B63] Yue L. Yin X. Hao F. Dong J. Ren X. Xu O. (2020). Long noncoding RNA Linc00632 inhibits interleukin-13-induced inflammatory cytokine and mucus production in nasal epithelial cells. J. Innate Immun. 12 (1), 116–128. 10.1159/000500420 31315126 PMC6959101

[B64] Zhang B. Orning P. Lehman J. W. Dinis A. Torres-Ulloa L. Elling R. (2024). Raver1 links Ripk1 RNA splicing to caspase-8-mediated pyroptotic cell death, inflammation, and pathogen resistance. bioRxiv. 10.1101/2024.11.27.625707 PMC11848402 39946533

[B65] Zhou J. Sun Y. Huang W. Ye K. (2021). Altered blood cell traits underlie a major genetic locus of severe COVID-19. J. Gerontol. A Biol. Sci. Med. Sci. 76 (8), e147–e154. 10.1093/gerona/glab035 33530099 PMC7929197

[B66] Zhu J. Yamane H. Cote-Sierra J. Guo L. Paul W. E. (2006). GATA-3 promotes Th2 responses through three different mechanisms: induction of Th2 cytokine production, selective growth of Th2 cells and inhibition of Th1 cell-specific factors. Cell Res. 16 (1), 3–10. 10.1038/sj.cr.7310002 16467870

[B67] Zoufaly A. Poglitsch M. Aberle J. H. Hoepler W. Seitz T. Traugott M. (2020). Human recombinant soluble ACE2 in severe COVID-19. Lancet Respir. Med. 8 (11), 1154–1158. 10.1016/s2213-2600(20)30418-5 33131609 PMC7515587

