# Supplementary material for: Exploring genetic loci linked to COVID-19 severity and immune response through multi-trait GWAS analyses
Source: Front Genet. 2025 Feb 17;16:1502839. doi: 10.3389/fgene.2025.1502839 (PMC11873281; doi:10.3389/fgene.2025.1502839)

Supplementary Figure 1.

Supplementary Figure 1. Heat maps showing patterns of cell-type-specific enrichments of SNP-heritability for genetically associated traits across 396 cell-type-specific annotations. Each checkered rectangle reflects the z-score, scaled by traits. Red indicates enrichment, blue indicates depletion. Deeper color represents stronger magnitude of effects. Asterisks represent statistical significance withstanding multiple correction. The category of cell-types is color coded to the left. A) DNase (DNase I hypersensitive sites), B) H3K27ac, C) H3K36me3, D) H3K4me1, E) H3K4me3, and F) H3K9ac.

A) DNase


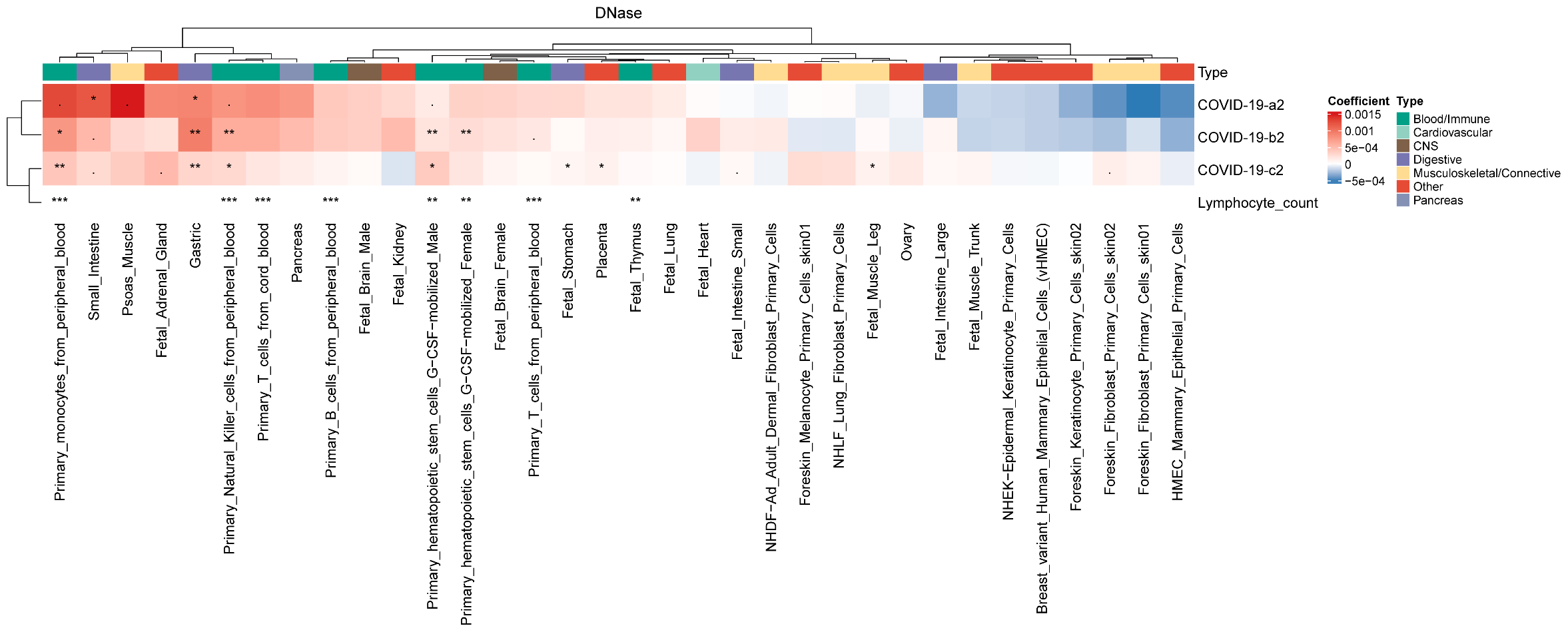


B) H3K27ac


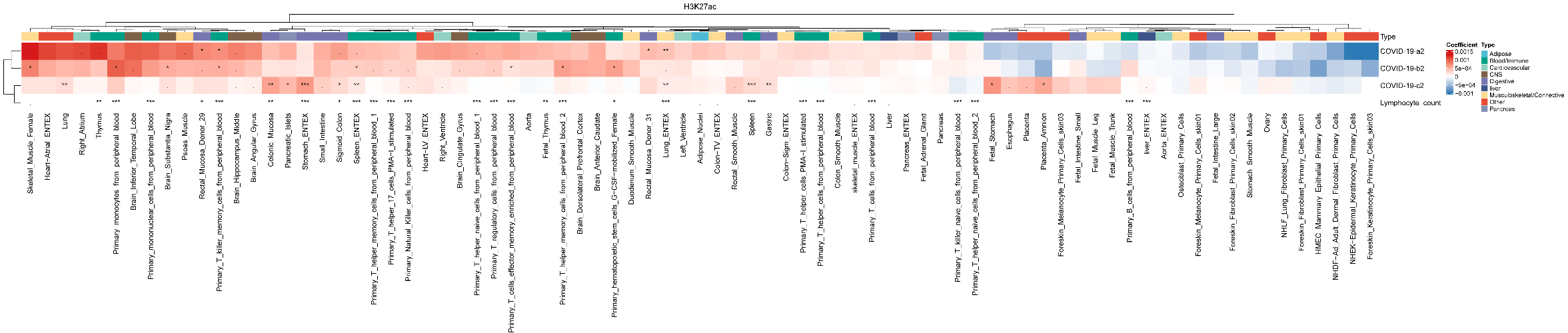


C) H3K36me3


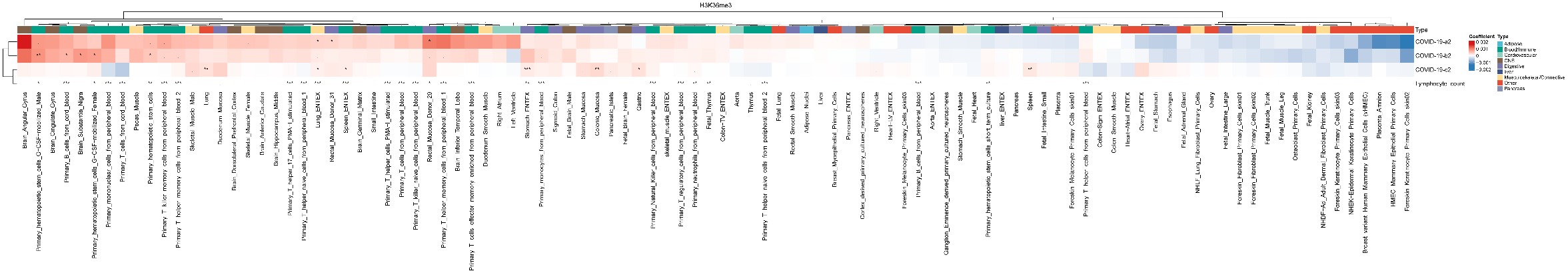


D) H3K4me1


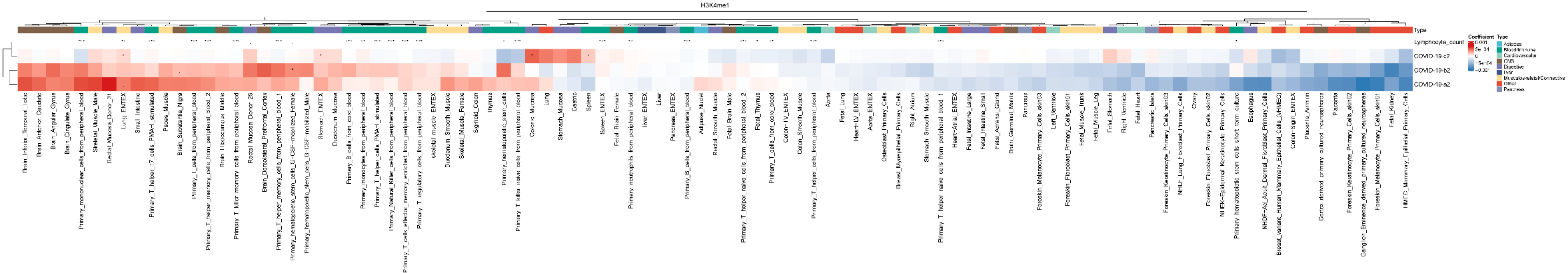


E) H3K4me3


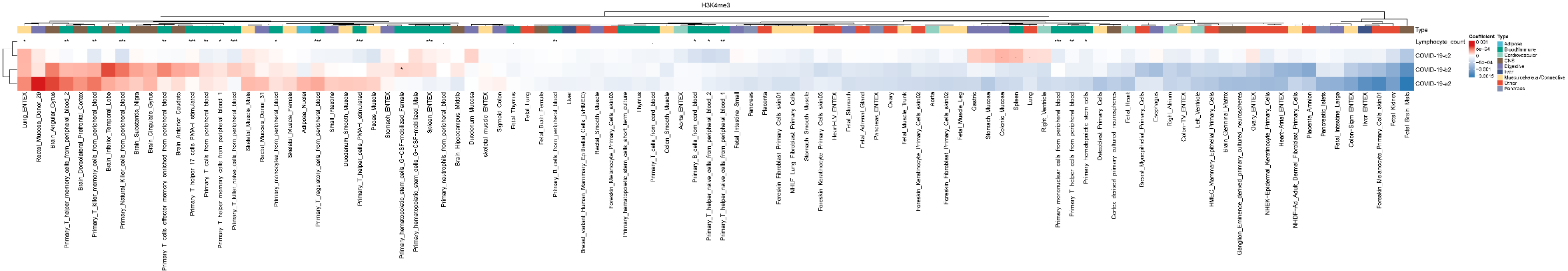


F) H3K9ac


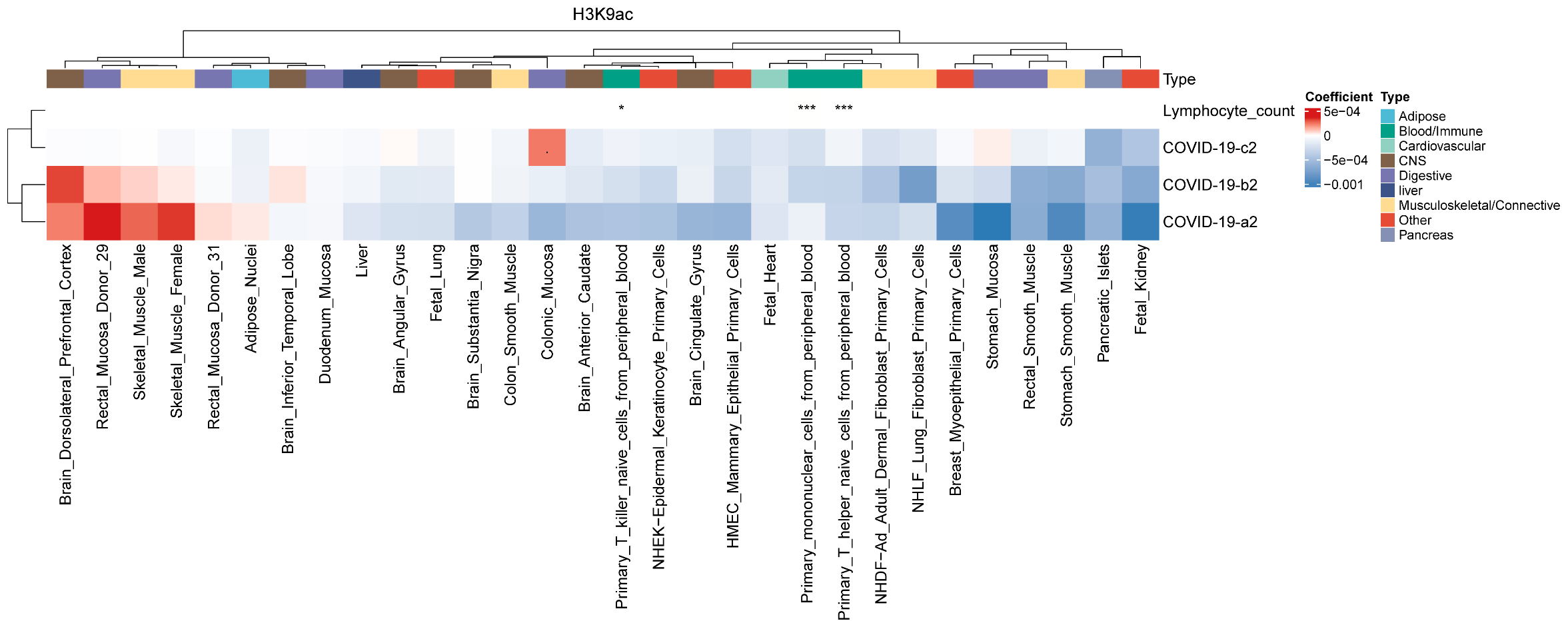


Supplementary Figure 2. QQ plots

The expected *P*-value inflation coefficient should be 1; when the actual inflation coefficient deviates from 1, it indicates that the phenomenon of population stratification may be more severe, leading to an increase in false positive results. The inflation coefficients (lambda): (A) COVID-19-a2.txt: 1.055; (B) COVID-19-b2.txt: 1.067; (C) COVID-19-c2.txt: 1.078; (D) Lymphocyte-count.txt: 1.14; (E) COVID-19-a2-mtag-trait-1.txt: 1.048; (F) COVID-19-b2-mtag-trait-1.txt: 1.041; (G) COVID-19-c2-mtag-trait-1.txt: 1.023.

(A) COVID-19-a2.txt: 1.055


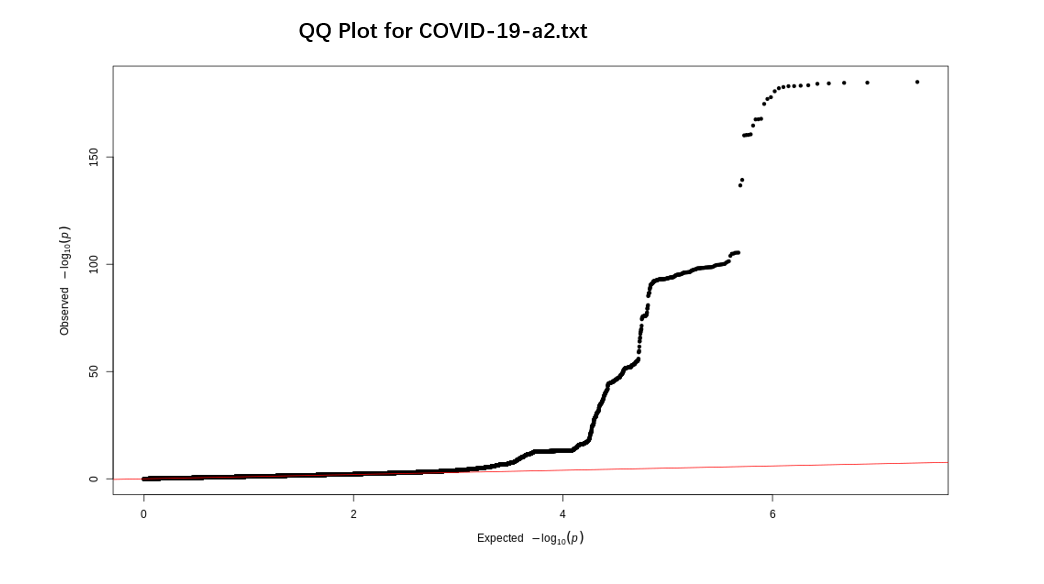


(B) COVID-19-b2.txt: 1.067


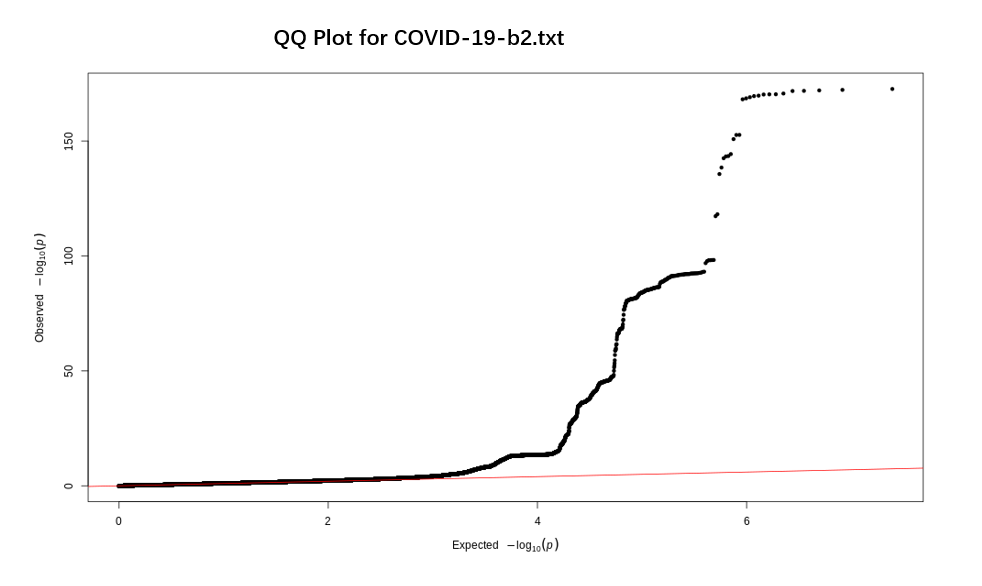


(C) COVID-19-c2.txt: 1.078


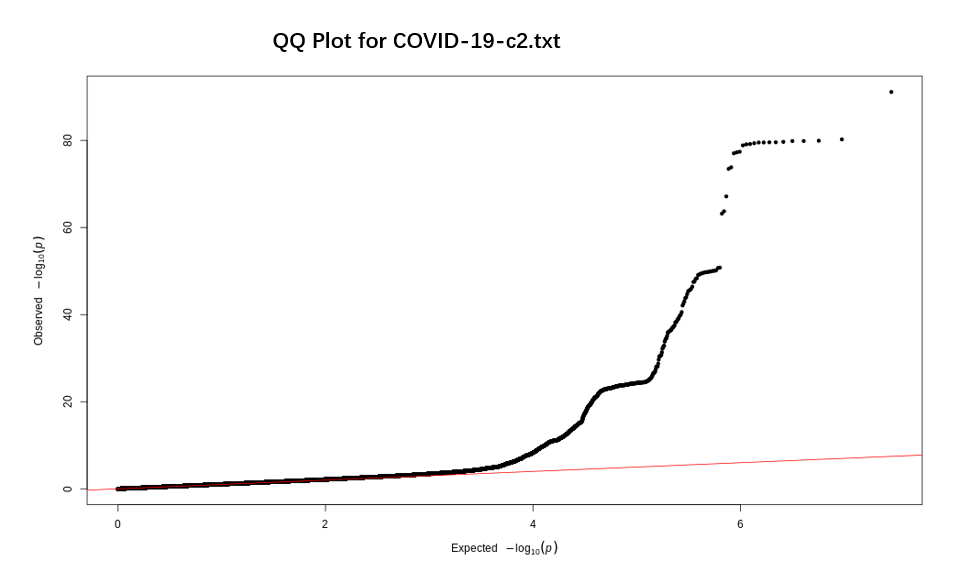


(D) Lymphocyte_count.txt: 1.14


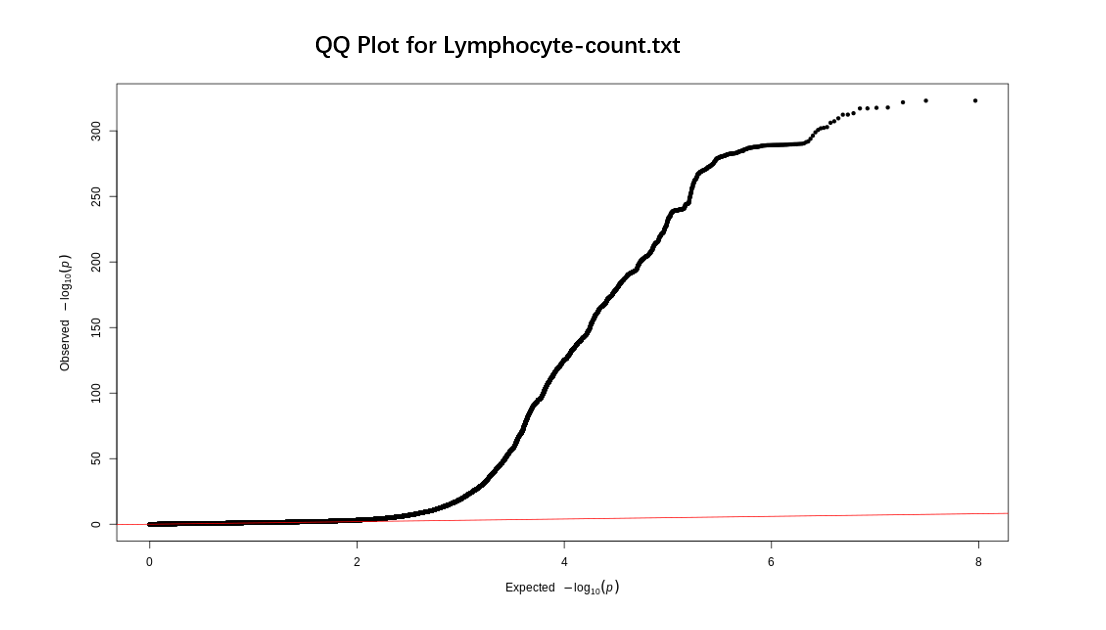


(E) COVID-19-a2-mtag-trait-1.txt: 1.048


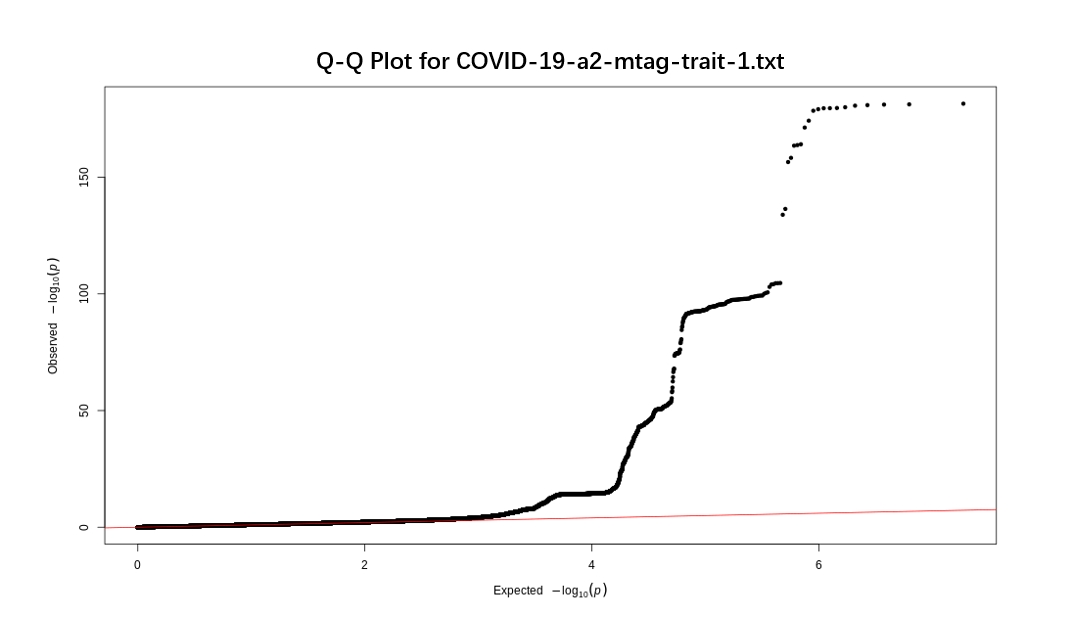


(F) COVID-19-b2-mtag-trait-1.txt: 1.041


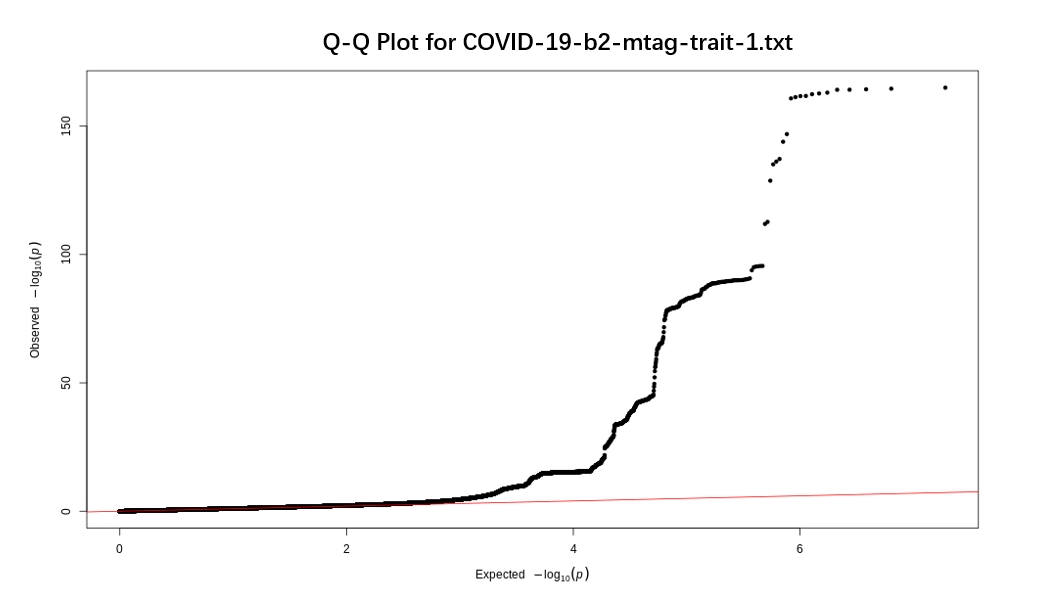


(G) COVID-19-c2-mtag-trait-1.txt: 1.023


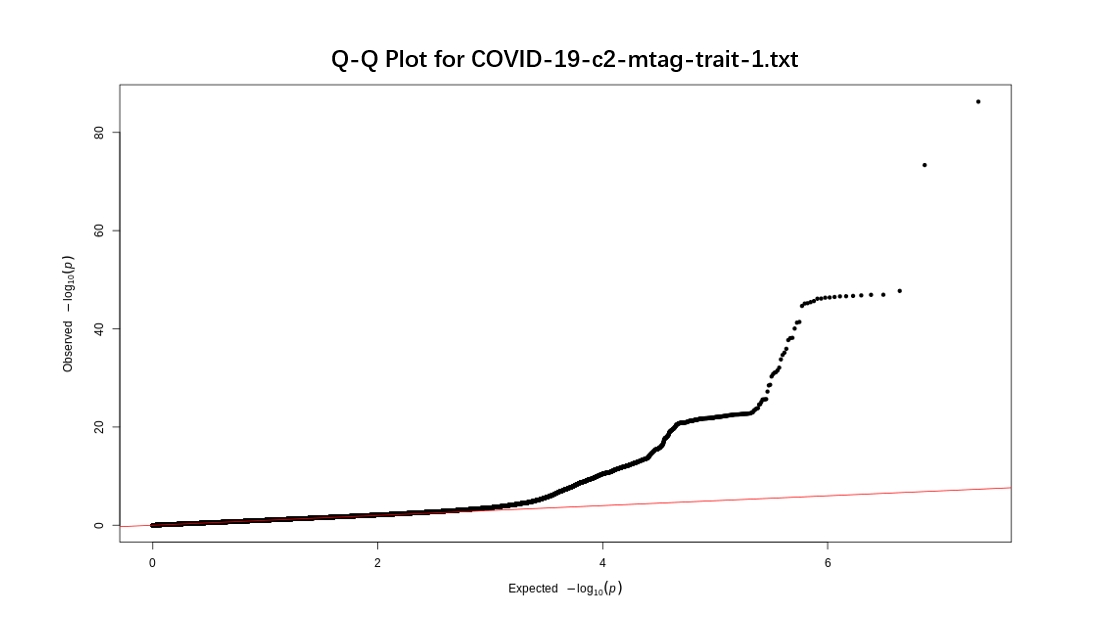


Supplementary Figure 3. Manhattan plots

(A) Manhattan Plot for COVID-19-a2.txt, (B) Manhattan Plot for COVID-19-b2.txt, (C) Manhattan Plot for COVID-19-c2.txt, (D) Manhattan Plot for Lymphocyte-count.txt

(E)Manhattan Plot for COVID-19-a2-mtag-trait-1.txt, (F) Manhattan Plot for COVID-19-b2-mtag-trait-1.txt, (G) Manhattan Plot for COVID-19-c2-mtag-trait-1.txt

(A)


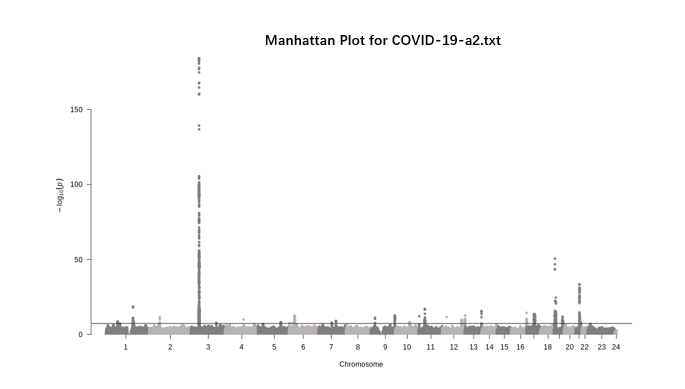


(B)


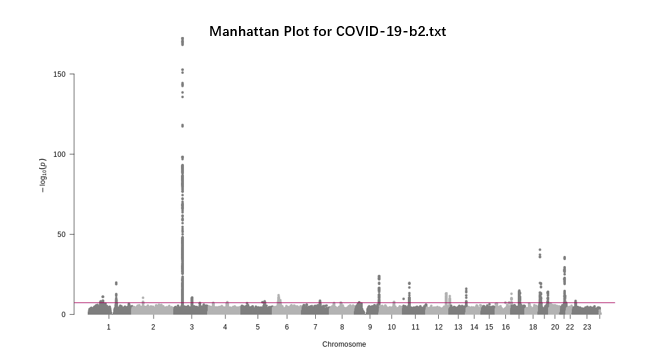


(C)


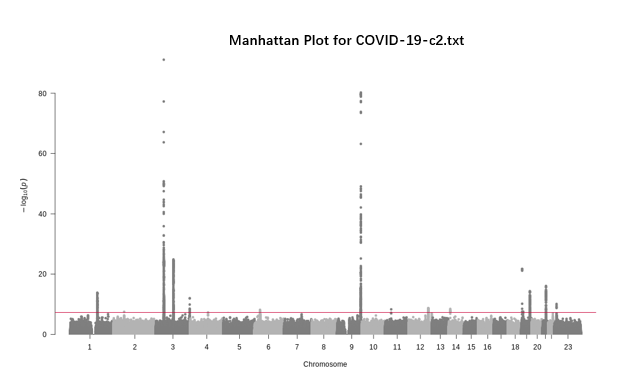


(D)


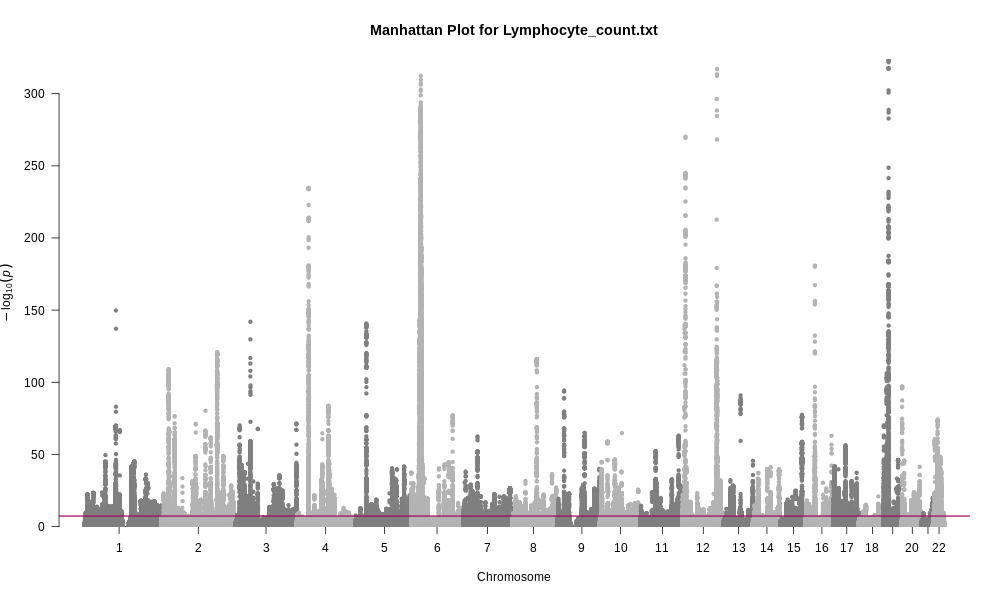


(E)


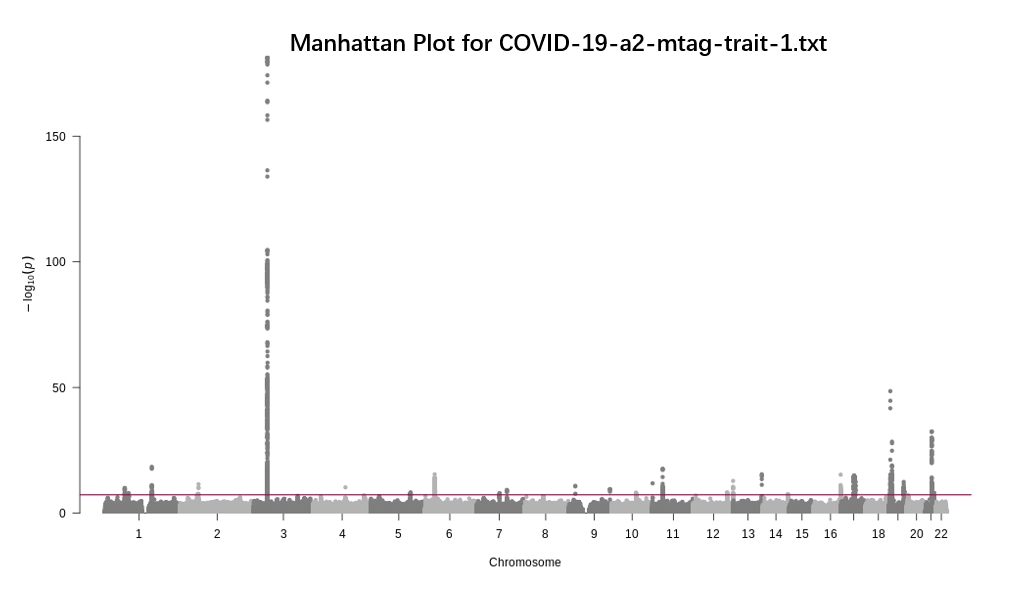


(F)


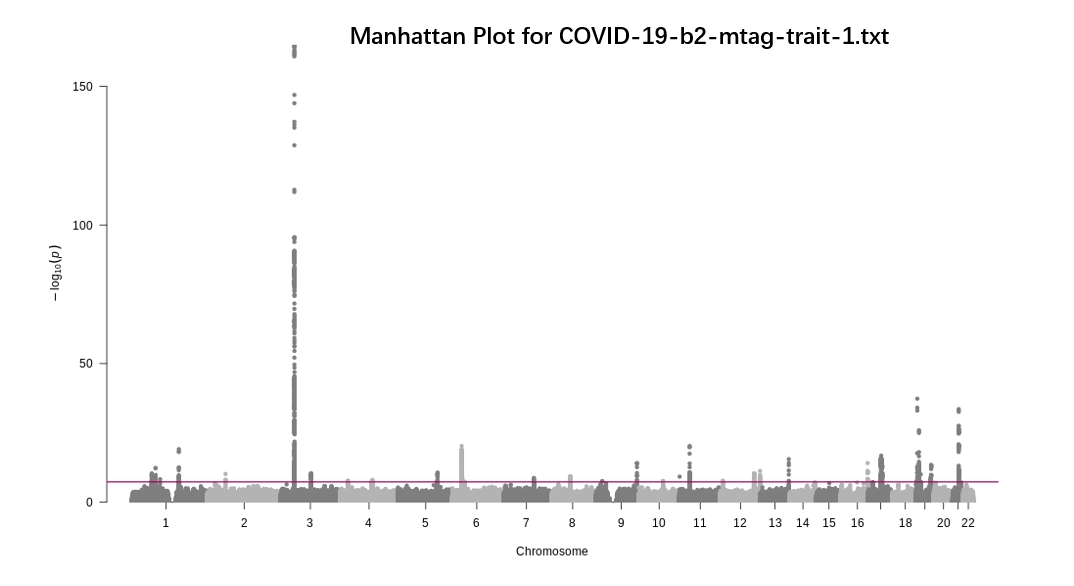


(G)


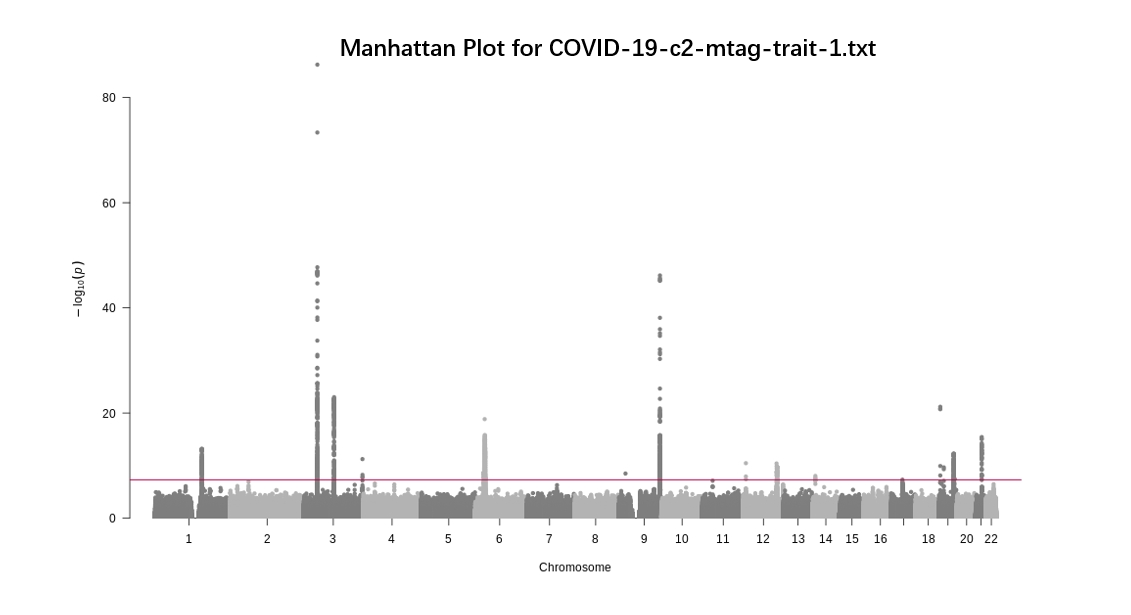

Supplement: Supplementary file 1 [file DataSheet1.docx]
